# Segmentation of multi-regional skeletal muscle in abdominal CT image for cirrhotic sarcopenia diagnosis

**DOI:** 10.3389/fnins.2023.1203823

**Published:** 2023-06-05

**Authors:** Genshen Song, Ji Zhou, Kang Wang, Demin Yao, Shiyao Chen, Yonghong Shi

**Affiliations:** ^1^Digital Medical Research Center, School of Basic Medical Sciences, Fudan University, Shanghai, China; ^2^Shanghai Key Laboratory of Medical Imaging Computing and Computer Assisted Intervention, Shanghai, China; ^3^Department of Gastroenterology and Hepatology, Zhongshan Hospital, Fudan University, Shanghai, China; ^4^Academy for Engineering & Technology, Fudan University, Shanghai, China

**Keywords:** cirrhotic sarcopenia, skeletal muscle segmentation, rectus abdominis, texture attention enhancement block, skeletal muscle index

## Abstract

**Background:**

Sarcopenia is generally diagnosed by the total area of skeletal muscle in the CT axial slice located in the third lumbar (L3) vertebra. However, patients with severe liver cirrhosis cannot accurately obtain the corresponding total skeletal muscle because their abdominal muscles are squeezed, which affects the diagnosis of sarcopenia.

**Purpose:**

This study proposes a novel lumbar skeletal muscle network to automatically segment multi-regional skeletal muscle from CT images, and explores the relationship between cirrhotic sarcopenia and each skeletal muscle region.

**Methods:**

This study utilizes the skeletal muscle characteristics of different spatial regions to improve the 2.5D U-Net enhanced by residual structure. Specifically, a 3D texture attention enhancement block is proposed to tackle the issue of blurred edges with similar intensities and poor segmentation between different skeletal muscle regions, which contains skeletal muscle shape and muscle fibre texture to spatially constrain the integrity of skeletal muscle region and alleviate the difficulty of identifying muscle boundaries in axial slices. Subsequentially, a 3D encoding branch is constructed in conjunction with a 2.5D U-Net, which segments the lumbar skeletal muscle in multiple L3-related axial CT slices into four regions. Furthermore, the diagnostic cut-off values of the L3 skeletal muscle index (L3SMI) are investigated for identifying cirrhotic sarcopenia in four muscle regions segmented from CT images of 98 patients with liver cirrhosis.

**Results:**

Our method is evaluated on 317 CT images using the five-fold cross-validation method. For the four skeletal muscle regions segmented in the images from the independent test set, the avg. DSC is 0.937 and the avg. surface distance is 0.558 mm. For sarcopenia diagnosis in 98 patients with liver cirrhosis, the cut-off values of Rectus Abdominis, Right Psoas, Left Psoas, and Paravertebral are 16.67, 4.14, 3.76, and 13.20 cm^2^/m^2^ in females, and 22.51, 5.84, 6.10, and 17.28 cm^2^/m^2^ in males, respectively.

**Conclusion:**

The proposed method can segment four skeletal muscle regions related to the L3 vertebra with high accuracy. Furthermore, the analysis shows that the Rectus Abdominis region can be used to assist in the diagnosis of sarcopenia when the total muscle is not available.

## 1. Introduction

Sarcopenia is a pathological decrease in skeletal muscle, including primary sarcopenia and secondary sarcopenia. Primary sarcopenia is the aging and atrophy of skeletal muscle with age, which is related to the aging process of humans. And secondary sarcopenia is caused by poor dietary intake, malnutrition and chronic diseases such as cirrhosis of the liver (Bauer et al., 2019). Sarcopenia is a common complication in patients with liver cirrhosis, characterized by the loss of muscle strength and mass. According to statistics (Xiao et al., 2019), as many as 7 million people in China suffer from cirrhosis, accounting for 0.5% of the total population. The prevalence of sarcopenia in cirrhotic patients is between 40% and 70% due to metabolic abnormalities resulting from decreased liver function (Cao et al., 2017). Study (Tantai et al., 2022) shows that cirrhotic sarcopenia increases the risk of falls, fractures, decreased quality of life, or acute-on-chronic liver failure in patients with cirrhosis. Sarcopenia is significantly associated with morbidity and mortality in cirrhotic patients (Hanai et al., 2015) and is an independent predictor of survival in patients with cirrhosis (Kim et al., 2017). Therefore, early and accurate diagnosis of sarcopenia is helpful for the clinical treatment and management of liver cirrhosis patients.

Sarcopenia is generally diagnosed by the third lumbar skeletal muscle index (L3SMI). L3SMI is defined by measuring the skeletal muscle area in the axial CT slice of the third lumbar (L3) vertebra, and then calculating the ratio of cross-sectional muscle area to the square of body height. For diagnosing patients with cirrhotic sarcopenia, the L3SMI’s cut-off values are 50 cm^2^/m^2^ in males and 39 cm^2^/m^2^ in females (Carey et al., 2017). However, in some diseases, it would not be enough to only measure these muscles. For example, parts of the abdominal muscles of patients with severe ascites may be severely squeezed; or the progression of myosteatosis varies in different muscle regions in nonalcoholic fatty liver disease. A recent study also explored the sarcopenia defined by different muscle groups such as total skeletal muscle, psoas major muscle, and rectus abdominis muscle as a prognostic factor for patients with advanced hepatocellular carcinoma (Wu et al., 2021). This shows that in the diagnosis of cirrhotic sarcopenia, considering the effect of disease on muscle in different regions, partitioning skeletal muscle regions and analyzing each muscle region separately may be a useful supplement to the analysis of the total skeletal muscle.

**Figure 1:**
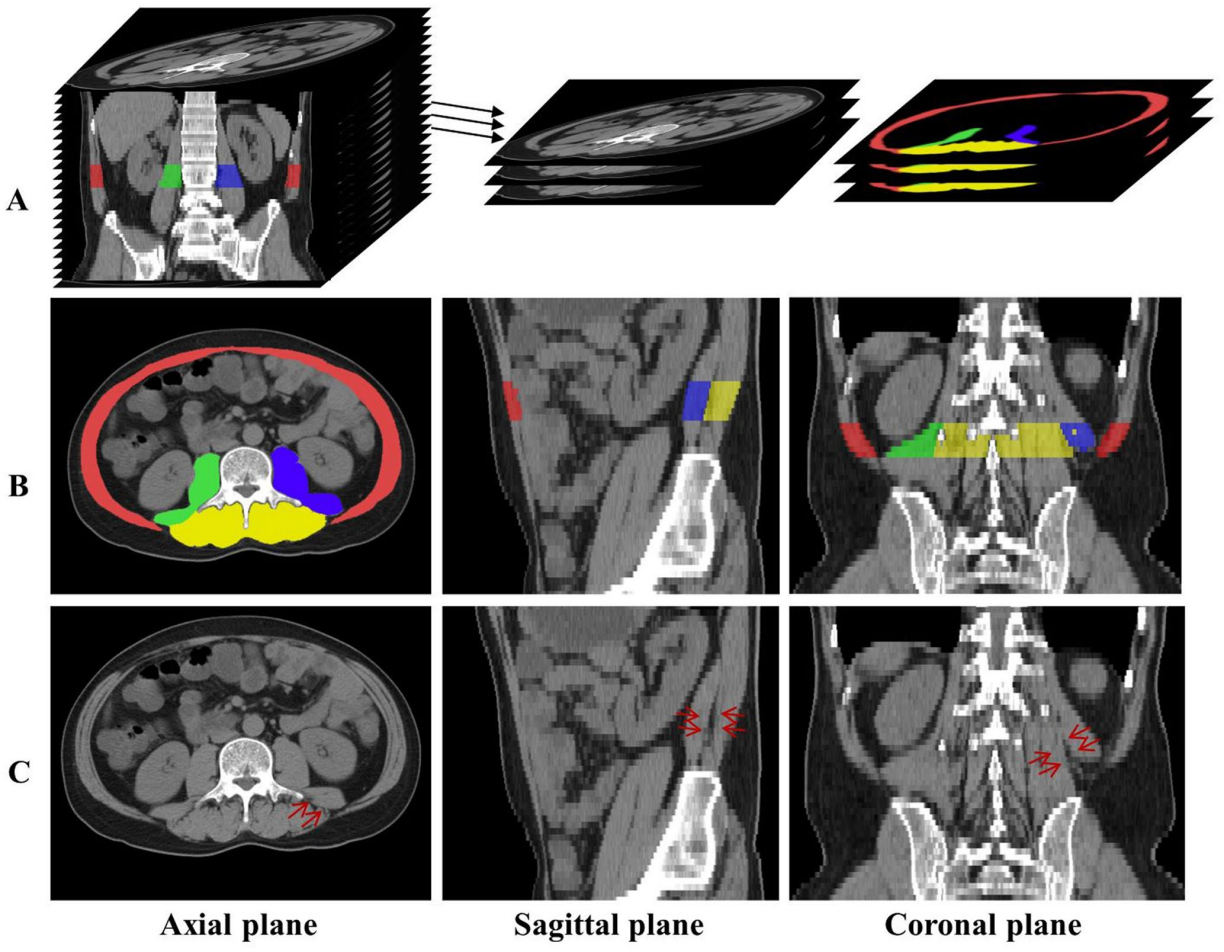
**(A)** The axial CT slices related to L3 are labeled and extracted. **(B)** The distribution of the four skeletal muscle regions is displayed in axial, sagittal, and coronal planes, and red, green, blue, and yellow represent the labels of Rectus Abdominis, Right Psoas, Left Psoas, and Paravertebral, respectively. **(C)** The red arrows indicate the skeletal muscle in the same location, and the skeletal muscles indistinguishable in the axial plane have distinct distinguishing features in the sagittal and coronal planes.

Therefore, this paper will study the multi-regional skeletal muscles from multiple L3-related CT slices. As shown in Figure 1, red, yellow, green, and blue represent the labels of Rectus Abdominis (the rectus abdominis, external oblique abdominis, internal oblique abdominis, and transversus abdominis at the anterior periphery of L3), Paravertebral (the paravertebral muscle groups such as the erector spine at the posterior part of L3), Right Psoas and Left Psoas (the psoas major, psoas minor, and psoas square on the right and left sides of L3) respectively. Once these skeletal muscle regions are segmented from the L3-related axial CT slices, they can efficiently assist in the diagnosis of sarcopenia.

However, there are various challenges in segmenting multiple skeletal muscle regions in abdominal or abdominopelvic CT images. As shown in Figure 1, there are obvious differences in the shape and size of different skeletal muscles; the boundaries between different skeletal muscle regions or between skeletal muscle and surrounding tissue are unclear or rough, such as the edges of the Right Psoas and Left Psoas in Figure 1; morphological differences of the same skeletal muscle region between different individuals affect segmentation; physiopathological conditions such as muscle fatty degeneration and muscle-reducing obesity affect muscle morphology and signal intensity in CT images; artifacts in CT images increase the difficulty of segmentation.

Deep Convolution Neural Network (CNN) (LeCun et al., 1998) is an effective model for muscle region segmentation in abdominal CT images, including Fully Convolutional Network (FCN) (Long et al., 2015) architecture and encoder–decoder-based models such as 2D U-Net (Ronneberger et al., 2015), 3D U-Net (Çiçek et al., 2016), and Swin-unet (Cao et al., 2023). For example, Dabiri et al. (2019) used FCN and 2D U-Net to segment skeletal muscles in L3- or L4-related CT slices for body composition analysis. Castiglione et al. (2021) and Dabiri et al. (2020) firstly automatically located the axial slice at the L3 centroid from a whole-body or partial-body CT image, and then used 2D U-Net–based models to segment body components, such as skeletal muscle. Park et al. (2020) developed and validated an FCN-based system to analyze skeletal muscles in the axial CT images at the inferior endplate of the L3. Blanc-Durand et al. (2020) used CNN to predict the muscle surface from the axial CT slices related to L3. And Weston et al. (2020) used U-Net variant architecture to segment muscles and other tissues in the abdominopelvic CT images. However, these methods only considered the total skeletal muscle segmentation but did not pay attention to different muscle region segmentation. The relationship between the total skeletal muscle and the diagnosis of sarcopenia can be obtained, but the diagnostic effectiveness of muscles in each region cannot be analyzed.

Recent studies have gradually focused on the segmentation of multiple skeletal muscle regions. Burns et al. (2020) used 2D U-Net-based model to automatically segment multiple muscle groups in the L3- and L4-related axial CT slices to detect central sarcopenia. Huang et al. (2020) used BS-ESNet to automatically segment paravertebral muscles in axial MRI slices at different spine levels. Barnard et al. (2019) used 2D U-Net based model to automatically segment the left paraspinal muscle in the axial CT slice at the twelfth thoracic vertebra. Although these methods focused on muscle segmentation in different regions, they did not pay attention to the multi-regional analysis in multiple axial CT slices related to L3. And they did not explore the relationship between cirrhotic sarcopenia and each skeletal muscle region.

Therefore, the study presents the method to accurately segment multiple skeletal muscle regions in the axial slices associated with the L3 vertebra, and then calculate the clinical indices and use them for the diagnosis of sarcopenia. L3SMI can usually be calculated from muscle regions segmented in two consecutive axial slices associated with the L3 vertebra, i.e., L3 middle and its adjacent lower slices (Wang et al., 2020), or one axial slice, i.e., L3 upper (Carey et al., 2017) or end slice (Li et al., 2020). However, recent studies demonstrated that the average difference of the skeletal muscle volume measurement was significantly lower than that of the corresponding region in a single CT slice by segmenting the entire abdominopelvic skeletal muscle (Borrelli et al., 2021). Inspired by this, the study uses the average cross-sectional area of the total skeletal muscle volume corresponding to the L3 vertebra to calculate a more reasonable skeletal muscle index. Furthermore, the relationship between each regional skeletal muscle and L3SMI is also investigated for sarcopenia diagnosis.

## 2. Materials and methods

### 2.1. Data description

This study used abdominal or abdominopelvic CT images of 317 patients from Zhongshan Hospital affiliated to Fudan University in Shanghai, China, including 216 cirrhotic patients and 101 non-cirrhotic patients. And height and gender of 98 patients in the cirrhosis group were also collected to analyze the relationship between sarcopenia and each skeletal muscle region. According to the diagnostic criteria of cirrhotic sarcopenia (Carey et al., 2017), there were 54 patients with sarcopenia, 43 of which were male and 11 females, and 44 patients with non-sarcopenia, 21 of which were male and 23 females. The mean age of the patients was 57 years old.

The imaging parameters for abdominal or abdominopelvic CT scans are as follows: the in-plane spacing is between 0.562 mm × 0.562 mm and 0.888 mm × 0.888 mm; the slice thickness is 5.0 mm; the image acquisition matrix is 512 × 512; and the number of L3 related axial slices are between 4 and 8.

Experienced clinicians manually labeled the skeletal muscle regions in all L3-related axial CT slices. According to muscle type and distribution, four skeletal muscle regions in the axial, sagittal, and coronal planes are obtained and shown in rows A and B of Figure 1. Here, red, green, blue, and yellow represent the label of Rectus Abdominis, Right Psoas, Left Psoas, and Paravertebral, respectively.

### 2.2. L3 localization and image preprocessing

Abdominal or abdominopelvic CT images contain many abdominal and lumbar regions, so it is necessary to accurately locate the L3 vertebra. This can be achieved by our developed method of automatic localization and identification of vertebra in spine CT images (Qin et al., 2021), which is further checked and confirmed by the clinician. Once the L3 was successfully detected, all axial slices related to L3 can be extracted, totaling about 4 to 8 slices, as shown in row A of Figure 1.

For all L3-related axial slices extracted from each CT image, if the number of the slices was less than 8, zero-padding was performed along the axial direction, so that the number of L3-related axial slices of all CT images was equal. Finally, the image block composed of L3-related slices was represented by a tensor with size 8 × 512 × 512 (depth × height × width), which was convenient for inputting the network and extracting the axial space feature. The extracted slices were processed by intensity normalization. Considering the fact that the minimum and maximum Hounsfield Unit (HU) values are varied among all CT images, in order to obtain better image contrast, the full range of HU values of each image was mapped to [0, 1].

### 2.3. Skeletal muscle segmentation network

**Figure 2:**
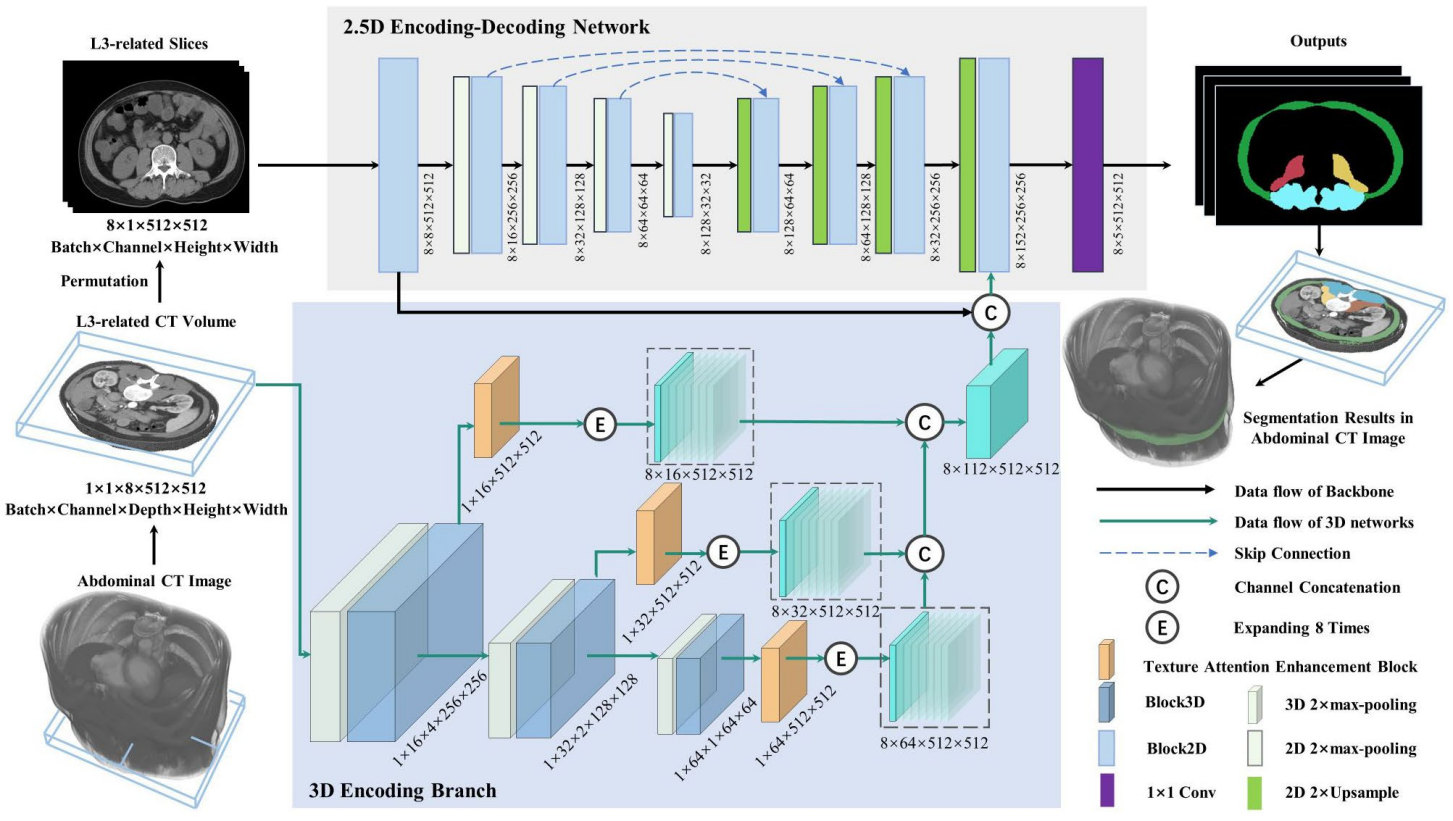
Illustration of the lumbar skeletal muscle segmentation network (LSMU-Net). LSMU-Net containing two parts, i.e., 2.5D Encoding-Decoding Network and 3D Encoding Branch. The dimension representation pattern is batch × channel × height × width in the 2.5D network, whereas batch × channel × depth × height × width in the 3D branch.

Figure 2 depicts the lumbar skeletal muscle segmentation network (LSMU-Net for short). The input of the network is the multiple L3-related axial slices of the abdominal CT images, and the outputs are the labels of the four skeletal muscle regions. The network mainly consists of two hybrid architectures, i.e., a 2.5D encoding–decoding network improved by residual structure, and a 3D encoding branch that enhances the spatial texture information. Specifically, the dedicated texture attention enhancement block is utilized to discern the blurred skeletal muscle boundaries in the 2D axial image shown in row C of Figure 1 from the 3D image space. The details are described as following.

#### 2.3.1. 2.5D encoding-decoding network

The 2.5D network, which is composed of the encoding and decoding branches connected by skip channel connections, is used to implement segmentation in the axial CT slice image. Here, although the 2.5D branch uses 2D convolution kernel, the input of the network consists of CT volumes with multiple slices. In particularly, to adapt to the 2.5D network, slices of the input volume are squeezed into a batch, so that a volume represented by a tensor with size 1 × 1 × 8 × 512 × 512 (batch × channel × depth × height × width) is squeezed and processed by a dimensional permutation to the size of 8 × 1 × 512 × 512 (batch × channel × height × width). Here, 8 originally denotes the depth dimension of the volume and then the number of image batch. The batch of the permutated slices is fed into the 2.5D encoding branch containing 5 successive Block2D modules with 4 stages of 2× max-pooling layer, and then goes through the decoding branch with 4 stages of 2× Upsample and Block2D module to obtain the hierarchical feature map at each stage. The features of the corresponding layer are concatenated in the channel dimension. In the last layer of the decoding branch, the feature map is restored to the same size as the input image and fused with the output features of both the 3D encoding branch and the channel connection in the channel direction, then the feature map represented by a tensor with size 8 × 152 × 512 × 512 is obtained. Finally, a 1 × 1 convolution layer is deployed to obtain the prediction maps of 5 categories represented by a tensor with size 8 × 5 × 512 × 512 as the final outputs (4 regions of skeletal muscles and background).

#### 2.3.2. 3D encoding branch

This is the contextual feature extraction network of the volumetric region composed of multiple L3-related axial slices. The network consists of 3 layers. Each layer of the 3D encoding branch is composed of the max-pooling layer, a Block3D module, and a texture attention enhancement block. The output feature map of the Block3D is halved by down-sampling using a max-pooling operation. The obtained feature maps are transferred to the next layer, simultaneously enhanced by the texture attention enhancement block, then restored to the original image size, and finally expanded 8 times by duplication operation for connecting with the output feature of the 2D decoder branch in the channel dimension. In the study, the input is an L3-related volumetric image represented by a tensor with size 1 × 1 × 8 × 512 × 512. After multiple layers of extracted features are concatenated to form 3D hierarchical features, the tensor size is 8 × 112 × 512 × 512 (batch × channel × height × width). Furthermore, the channel connection operation is performed with the feature of the last layer of the 2.5D network.

**Figure 3:**
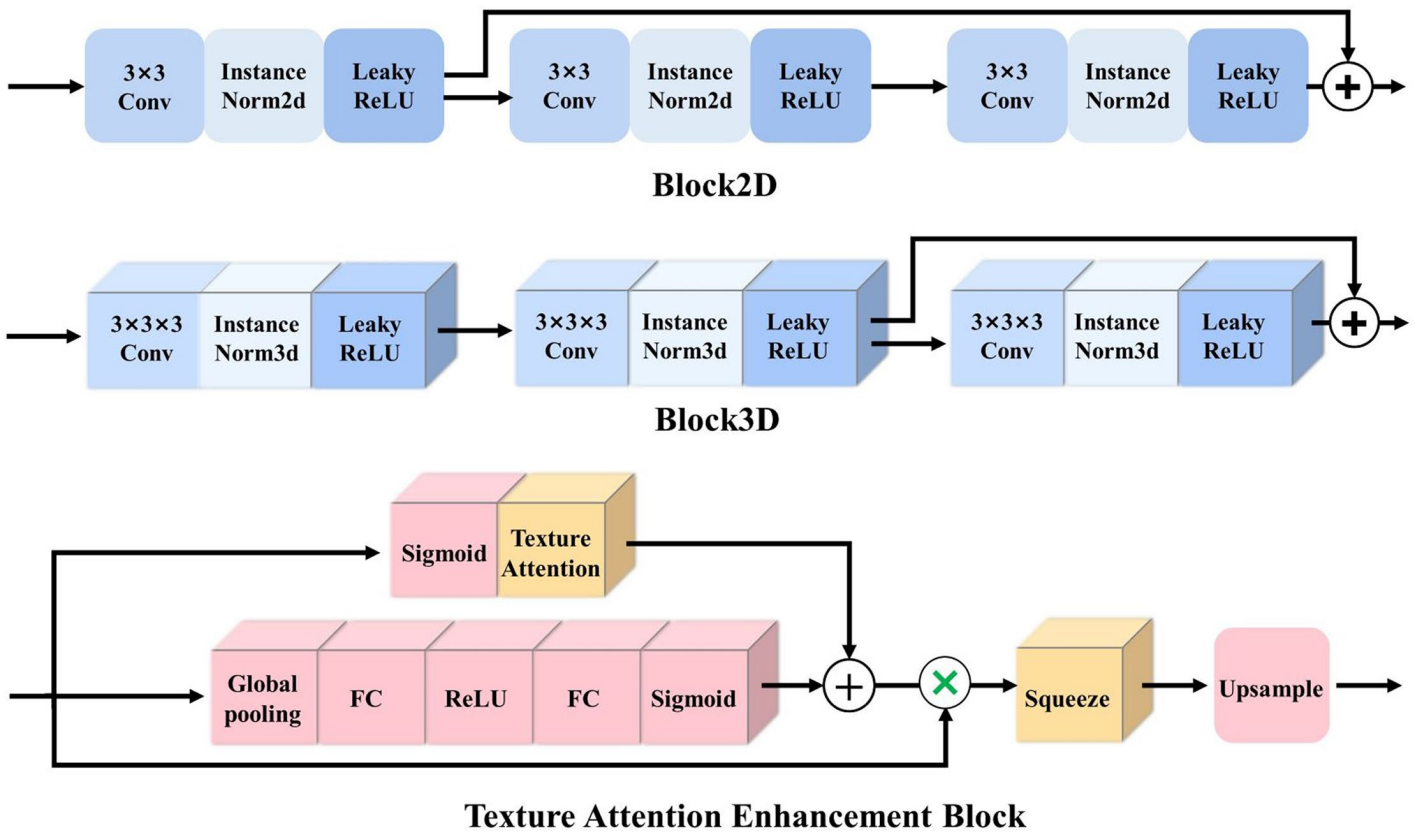
Illustration of the basic residual structures of 2.5D and 3D networks, i.e., Block2D and Block3D, as well as Texture Attention Enhancement Block.

**Block2D and Block3D**: as the basic structure of the LSMU-Net, Block2D and Block3D take the residual structure of 2D ResU-Net (He et al., 2016) as a reference, but they also have differences. First, the convolutional layers are cascaded with InstanceNorm and LeakyReLU to form the basic blocks; subsequently, three groups of basic blocks are cascaded and jump-connected to form Block2D or Block3D with residual structures, respectively, as shown in Figure 3. Block2D has a 3 × 3 convolutional kernel and Block3D has a 3 × 3 × 3 kernel. These two structural blocks do not change the number of channels, but can effectively deepen the model, facilitating finer edge feature extraction and providing better correction for skeletal muscle refinement. The down-sampling process of the 3D branch contains more trainable features, which requires more convolutional layers to extract spatial information. Therefore, the designs of residual connections in Block2D and Block3D are different, with more convolutional layers in Block3D so that spatial information and 3D structural characteristics can be sufficiently propagated and utilized in the whole network.

Here, it should be noted that the study takes the 2.5D network as the backbone structure. The initial reason is that the number of L3 axial CT slices is small, which limits multiple down-sampling of the 3D network. And the studies (Liu et al., 2017; Isensee et al., 2021) shows that conventional 3D segmentation methods may deteriorate the performance in the anisotropic medical image, and anisotropic convolution on specific planes with better resolution and more appearance features may also improve the accuracy (Jia et al., 2022). As shown in row C of Figure 1, the red arrows indicate the same position of the axial, sagittal, and coronal planes, and the skeletal muscles that cannot be distinguished in the axial plane have distinct characteristics in the sagittal and coronal planes. The 3D encoding branch precisely extracts the spatial context information (shown by the red arrows) lost in the 2.5D network, and the fusion of these features enables the 2.5D network to refine the edges of the skeletal muscle region from the shape of the muscle fiber bundle, improving the segmentation performance. And the studies (Meyer et al., 2021) also shows that the ensemble of 2.5D and 3D network does improve the accuracy in 3D medical image segmentation. Finally, the training time is reduced because the number of parameters in the 2.5D network is less than that of the 3D network.

#### 2.3.3. Texture attention enhancement block

To better integrate 3D features and 2.5D features, Zhou et al. (2019) simultaneously selected and trained four adjacent 2D slice images to complement the 3D features, then extracted the features from 2.5D and 3D branches, and fused them after attention enhancement. In this study, based on the standard Squeeze-and-excitation (SE) Block (Hu et al., 2020), a texture attention enhancement block is constructed to compress the channel feature in the 3D network and enhance the blurred edge regions, as shown in Figure 3. The features extracted by Block3D represented by a tensor with size channel × depth × height × width are fed into the attention block. Firstly, the global average pooling is carried out to obtain the feature map represented by a tensor with size channel × 1 × 1 × 1. Then it passes through two layers of a fully connected layer, in which the number of neurons in the first fully connected layer is channel/16 (following SE Block), and the second fully connected layer restores the original number of neurons. This operation increases the nonlinear processing and can fit the complex correlation between channels. Then the probability map is generated through the Sigmoid function. Secondly, the features extracted by Block3D are input into the Texture Attention Block after passing the Sigmoid function, and the pixel-level attention information is obtained through this operation. The proposed Texture Attention Block can increase the range of attention, as shown in Eq. (1).


(1) TextureAttention(x)=x(1−x)

where *x* represents the input probability map. This formula assigns a higher weight to the edge region whose probability is close to 0.5 and a lower weight to the area whose probability is far away from 0.5.

By adding the output feature of Eq. (1), the network no longer only pays attention to the middle part of the skeletal muscle but also enhances the edge refinement based on the shape constraints of the skeletal muscle fibre bundles. The texture attention enhancement block applied in the 3D branch is aim to calculate the weight of the corresponding pixel level and the weight of the channel at the same time, and combine the two. The utility of the texture attention enhancement block is based on the local information of the image, and more attention is paid to the skeletal muscle edge. The part of the skeletal muscle edge is given a high weight value through the pixel-level weight, and the background and the internal area of the skeletal muscle are set a small weight value. Finally, the channels are compressed by the Squeeze and Upsample block to restore the feature map represented by a tensor of original size 512 × 512 in the height and width directions for easy fusion with the 2.5D network.

#### 2.3.4. Loss function

For an input abdominal CT image, four skeletal muscle regions are segmented by combining the multi-class cross-entropy loss function, ${\mathrm{Loss}}_{\mathrm{ce}}$, and the dice loss function, ${\mathrm{Loss}}_{\mathrm{dice}}$. The calculation of these loss function is shown in Eqs. (2)–(4).


(2) $$\mathrm{Loss}={\mathrm{Loss}}_{\mathrm{ce}}+{\mathrm{Loss}}_{\mathrm{dice}}\;$$

(3) $${\mathrm{Loss}}_{\mathrm{ce}}=-\overset{C}{\underset{c=1}{\sum }}\overset{H\times W}{\underset{i=1}{\sum }}\omega_{c}y_{i}^{c}\log\left(\frac{e^{{\hat{y}}_{i}^{c}}}{\sum_{j=1}^{C}e^{j}}\right)$$

(4) $${\mathrm{Loss}}_{\mathrm{dice}}=\overset{C}{\underset{c=1}{\sum }}\omega_{c}\left(1-\frac{2\sum_{i=1}^{H\times W}y_{i}^{c}{\hat{y}}_{i}^{c}}{\sum_{i=1}^{H\times W}{\left(y_{i}^{c}\right)}^{2}+\sum_{i=1}^{H\times W}{\left({\hat{y}}_{i}^{c}\right)}^{2}}\right)$$

where *C* = 5 denotes the four skeletal muscle regions and the background. $\omega_{c}$ denotes the weight of region *c*. $y_{i}^{c}$ indicates the ground truth value of the $i^{\mathrm{th}}$ pixel which belongs to the $c^{\mathrm{th}}$ label. ${\hat{y}}_{i}^{c}$ denotes the predicted value of the $i^{\mathrm{th}}$ pixel which is predicted as the $c^{\mathrm{th}}$ label. *H* and *W* denote the height and width of the 2D axial CT image, respectively.

The sizes of the four skeletal muscle regions vary greatly, which means there is a class imbalance problem that may lead to the instability of the segmentation network. Therefore, during the training stage, it is necessary to punish the low confidence (such as Right Psoas and Left Psoas) prediction by setting the weight in the loss function. Specifically, the pixel proportions of the four skeletal muscle regions and background in the training images are counted, and then the regions with smaller proportions are set with large weights, and the regions with large proportions are set with small weights, as shown in Eq. (5).


(5) $$\omega_{c}=\{\begin{array}{ll} 1-\frac{N_{c}}{H\times W\times D}-0.2, & \;\mathrm{if}\;c=1\;\mathrm{or}\;c=4\\ 1-\frac{N_{c}}{H\times W\times D}, & \;\mathrm{if}\;c=2\;\mathrm{or}\;c=3 \end{array}$$

where *H*, *W*, and *D* denote the height, width, and depth of the training image and $N_{c}$ denotes the number of pixels counted in the $c^{\mathrm{th}}$label. As a result, the prior statistics of $\omega_{c}\;$ ensure the class equilibrium optimization of the loss function.

### 2.4. Training and testing parameter settings

The experiments were conducted on Ubuntu 18.04 operating system and PyTorch framework, configured with Intel^®^ Core^™^ i5-9600K (3.70 GHz × 6 CPUs), 64 GB RAM and RTX 3090 GPUs. The study was evaluated on the abdominal CT images of 317 patients, including 216 cirrhotic patients and 101 non-cirrhotic patients. Firstly, we randomly divided these data into training group (*n* = 252) and independent test group (*n* = 65). Cirrhotic images and non-cirrhotic images were evenly distributed in each group. Secondly, on the train group, we used the five-fold cross-validation method to evaluate the proposed algorithm. That is, we randomly divided all the sampled into five groups and used four groups for training and the left-out group for testing in each fold. Cirrhotic images and non-cirrhotic images were evenly distributed in each fold. And the Adam optimizer with a learning rate of 0.001 was used to execute for 30 epochs in each fold, and five models were obtained. Thirdly, the model with the best performance of five models was selected to run on the independent test group for the final inference.

### 2.5. Evaluation indicators

The evaluation metrics of our segmentation results are based on standard measures calculated from pixel-level confusion matrix, including Dice similarity coefficient (DSC) (Zou et al., 2004) and Sensitivity calculated from Eqs. (6) and (7), respectively.


(6) $$\mathrm{DSC}=\frac{2TP_{c}}{2TP_{c}+FP_{c}+FN_{c}}\;$$

(7) $$\mathrm{Sensitivity}=\frac{TP_{c}}{TP_{c}+FN_{c}}$$

where *c* denotes a category label. ${\mathrm{TP}}_{c}$ and ${\mathrm{TN}}_{c}$ denote the numbers of the true positive and the true negative pixels in the $c^{\mathrm{th}}$ skeletal muscle region, while ${\mathrm{FP}}_{c}$ and ${\mathrm{FN}}_{c}$ are the numbers of the false positive and the false negative pixels in that category, respectively.

Average symmetrical surface distance (ASSD) is the average Hausdorff distance between the outer surfaces of the segmentation result and the ground truth, calculated from Eqs. (8) and (9).


(8) $$d\left(v_{s},G_{c}\right)=\min_{v_{g}\in G_{c}}{\left\|v_{s}-v_{g}\right\|}_{2}$$

(9) $$\mathrm{ASSD}=\frac{1}{N_{G_{c}}+N_{S_{c}}}\left(\underset{s_{C}}{\sum }(\overset{N_{s_{c}}}{\underset{v_{s}=1}{\sum }}d\left(v_{s},G_{c}\right)+\overset{N_{G_{c}}}{\underset{v_{g}=1}{\sum }}d\left(v_{g},S_{c}\right))\;\right)$$

where $S_{c}$ and $G_{c}$ denote the surfaces of the segmentation and the ground truth of class *c*, respectively. The shortest distance from any voxel $v_{s}$ belonging to $\;S_{c}$ to$\;G_{c}$ is calculated in Eq. (8). ||·||_2_ represents the Euclidean Distance. $N_{S_{c}}$ and $N_{G_{c}}$ represent the number of voxels in the surfaces of the segmentation and the ground truth of class c, respectively. The unit for ASSD is a millimeter.

## 3. Experimental result

### 3.1. Ablation comparison experiments

To illustrate the overall structural validity of the proposed network, we reproduced 2D U-Net, 3D U-Net, 2D ResU-Net, and 3D ResU-Net (Lee et al., 2017) for comparison experiments. The normalization function and the activation function were InstanceNorm and LeakyReLU. In addition, since nnU-Net (Isensee et al., 2021) is an out-of-the-box representative of 3D U-Net, and has achieved the excellent results in several medical image segmentation tasks, in order to evaluate the performance of LSMU-Net, we used the latest code (nnU-Net V2, including 2D nnU-Net and 3D nnU-Net) from the official nnU-Net website^1^ to segment the same data set following the same cross-validation method. The cirrhosis dataset has previously been used for slice-based segmentation in literature (Liu et al., 2019), but that study only described the segmentation model and lacked a detailed description of the training set, the validation set, cross-validation, and DSC calculation of each dataset, so no comparison was made with it. It is worth noting that, in order to make a fair comparison, all the experiments in the study did not enhance the data, which indicates that the results may have the risk of overfitting.

Table 1 shows the DSC results of different methods in segmenting the four skeletal muscle regions of Rectus Abdominis, Right Psoas, Left Psoas and Paravertebral regions. It can be noted from rows 1–4 of Table 1 that the DSC values of the methods combining with the residual structure, namely 2D and 3D ResU-Net, are generally better than those of the corresponding 2D and 3D U-Net, respectively. Therefore, these structures were used in our network. The ablation experiments in rows 7 to 10 of Table 1 also show that the combination of residual structure in our method did improve the DSC values of all skeletal muscles. Rows 5 and 6 shows that the DSC values of the four skeletal muscle regions segmented by 2D and 3D nnU-Net. In particular, the DSC values by 3D nnU-Net are 0.948, 0.929, 0.922, and 0.954, respectively, and the corresponding values by our LSMU-Net are 0.943, 0.928, 0.922, and 0.957 in row 12. The segmentation performance of 3D nnU-Net is slightly higher than that of LSMU-Net in regions of Rectus Abdominis and Right Psoas. The DSC value of LSMU-Net is higher than that of 3D nnU-Net in Paravertebral. The DSC values of 3D nnU-Net and LSMU-Net are the same in the Left Psoas.

**Table 1:** LSMU-Net ablation comparison experiment shown on DSC in the independent test dataset.

	#	3D	AB	RS	W	Rectus abdominis	Right psoas	Left psoas	Paravertebral
3D U-Net	1					0.924 ± 0.001	0.914 ± 0.002	0.902 ± 0.004	0.946 ± 0.001
2D U-Net	2					0.936 ± 0.002	0.925 ± 0.002	0.915 ± 0.003	0.955 ± 0.000
3D ResU-Net	3			✓		0.925 ± 0.002	0.916 ± 0.003	0.909 ± 0.003	0.952 ± 0.001
2D ResU-Net	4			✓		0.940 ± 0.001	0.926 ± 0.002	0.918 ± 0.003	**0.957 ± 0.001**
3D nnU-Net	5					**0.948 ± 0.001**	**0.929 ± 0.002**	**0.922 ± 0.002**	0.954 ± 0.001
2D nnU-Net	6					0.946 ± 0.001	0.926 ± 0.003	0.916 ± 0.003	0.956 ± 0.000
LSMU-Net based	7			✓	✓	0.940 ± 0.001	0.925 ± 0.002	0.914 ± 0.002	0.954 ± 0.001
	8	✓		✓	✓	0.942 ± 0.001	0.926 ± 0.002	0.920 ± 0.003	**0.957 ± 0.001**
	9	✓	✓		✓	0.931 ± 0.001	0.916 ± 0.001	0.906 ± 0.003	0.950 ± 0.001
	10	✓	✓	✓		0.941 ± 0.001	0.927 ± 0.002	0.918 ± 0.003	**0.957 ± 0.001**
LSMU-Net + SE	11	✓		✓	✓	0.942 ± 0.001	0.926 ± 0.002	0.915 ± 0.003	0.956 ± 0.001
LSMU-Net	12	✓	✓	✓	✓	0.943 ± 0.001	0.928 ± 0.002	**0.922 ± 0.002**	**0.957 ± 0.001**

#, method number; 3D, 3D encoding branch; AB, attention block; RS, residual structure; W, weights. The bold value indicates that the method in row has achieved the best performance.

To evaluate the utility of each module, the ablation experiments of LSMU-Net were conducted from different perspectives while keeping the parameter settings unchanged. The DSC results of the four skeletal muscle regions are shown in rows 7 to 11 of Table 1. First, the residual module, 3D encoding branch, and texture attention enhancement block were removed from LSMU-Net, respectively. In row 7, there are no 3D branch nor attention block to enhance spatial information. Although the remaining 2.5D backbone network has the same residual structure as 2D ResU-Net, their convolutional layers are arranged differently because our Block2D has an additional layer of convolutional operation before the residual structure. In row 8, DSC increase in all four skeletal muscle regions by comparing to row 7 with the addition of the 3D branch without attention. As the 3D branch with attention block in the study mainly focuses on edge refinement to obtain more accurate skeletal muscle edges, which is more effective in improving the edges of skeletal muscles with small areas like the Right Psoas and Left Psoas. Thus, DSC could also be improved with the addition of only one 3D branch. In row 9, the network including Block2D and Block3D removes all residual structures compared to LSMU-Net. It can be seen that the decrease of DSC indicates that the residual structures in the network is useful for the segmentation of skeletal muscle. To illustrate the validity of the weights in the loss function, we removed the weights from LSMU-Net in row 10 and found that the DSCs of Rectus Abdominis, Right Psoas, and Left Psoas decrease compared with LSMU-Net. Secondly, row 11 shows the results of LSMU-Net using the SE block, which is slightly worse than the results of LSMU-Net using the texture attention enhancement block in row 12. This indicates that our texture attention enhancement block can improve the segmentation of fuzzy regions and optimize the performance of our method.

Table 2 shows the LSMU-Net ablation comparison experiment on the average index of four skeletal muscle regions in the independent test dataset. The prediction results of 3D nnU-Net are 0.938, 0.942, and 0.578 mm in terms of DSC, Sensitivity, and ASSD (mm), respectively. The corresponding results of LSMU-Net are 0.937, 0.944, and 0.558 mm, respectively. The ASSD of LSMU-Net is slightly lower than those of other networks.

**Table 2:** LSMU-Net ablation comparison experiment shown on the average index of four skeletal muscle regions in the independent test dataset.

	#	3D	AB	RS	W	DSC	Sensitivity	ASSD (mm)
3D U-Net	1					0.922 ± 0.002	0.918 ± 0.003	1.263 ± 19.103
2D U-Net	2					0.934 ± 0.002	0.941 ± 0.001	0.695 ± 2.261
3D ResU-Net	3			✓		0.925 ± 0.002	0.923 ± 0.003	0.691 ± 2.045
2D ResU-Net	4			✓		0.935 ± 0.002	0.942 ± 0.001	0.641 ± 0.845
3D nnU-Net	5					**0.938 ± 0.001**	0.942 ± 0.002	0.578 ± 1.187
2D nnU-Net	6					0.936 ± 0.002	0.941 ± 0.002	0.814 ± 6.055
LSMU-Net based	7			✓	✓	0.933 ± 0.002	0.937 ± 0.001	0.631 ± 0.785
	8	✓		✓	✓	0.936 ± 0.002	0.942 ± 0.001	0.695 ± 3.711
	9	✓	✓		✓	0.926 ± 0.002	0.922 ± 0.003	1.279 ± 12.220
	10	✓	✓	✓		0.936 ± 0.002	**0.947 ± 0.001**	0.677 ± 1.381
LSMU-Net + SE	11	✓		✓	✓	0.935 ± 0.002	0.939 ± 0.001	0.623 ± 0.881
LSMU-Net	12	✓	✓	✓	✓	0.937 ± 0.002	0.944 ± 0.001	**0.558 ± 0.715**

#, method number; 3D, 3D encoding branch; AB, attention block; RS, residual structure; W, weights. The bold value indicates that the method in row has achieved the best performance.

### 3.2. Quantitative segmentation results

Our LSMU-Net method was used to segment the four skeletal muscle regions in the CT image. Table 3 shows the accuracy of the segmentation results for the independent test dataset. For the four skeletal muscle regions, DSC reached above 0.92, and Sensitivity exceeded 0.93. Our method achieved the best segmentation results for the Paravertebral muscles, which were easy to segment because of their large area and concentration near the L3 vertebra. However, the skeletal muscles represented by Right Psoas and Left Psoas are very small, so the corresponding metrics are low, which makes the average values of the corresponding muscles less than those of the Paravertebral muscles.

**Table 3:** DSC, sensitivity, and ASSD (mm) of four skeletal muscle regions of the CT image in the independent test dataset segmented by our LSMU-Net.

	DSC	Sensitivity	ASSD (mm)
Rectus abdominis	0.943 ± 0.001	0.943 ± 0.002	0.431 ± 0.045
Right psoas	0.928 ± 0.002	0.941 ± 0.001	0.689 ± 1.299
Left psoas	0.922 ± 0.002	0.938 ± 0.001	0.701 ± 1.409
Paravertebral	0.957 ± 0.001	0.953 ± 0.001	0.410 ± 0.030
Average	0.937 ± 0.002	0.944 ± 0.001	0.558 ± 0.715

Table 3 also shows the average surface distance error of the four skeletal muscle regions. The Paravertebral muscle had the smallest ASSD of 0.410 mm; while the Left Psoas muscle had the largest ASSD at 0.689 mm. The average ASSD for all skeletal muscles reached 0.558 mm.

### 3.3. Qualitative segmentation results

**Figure 4:**
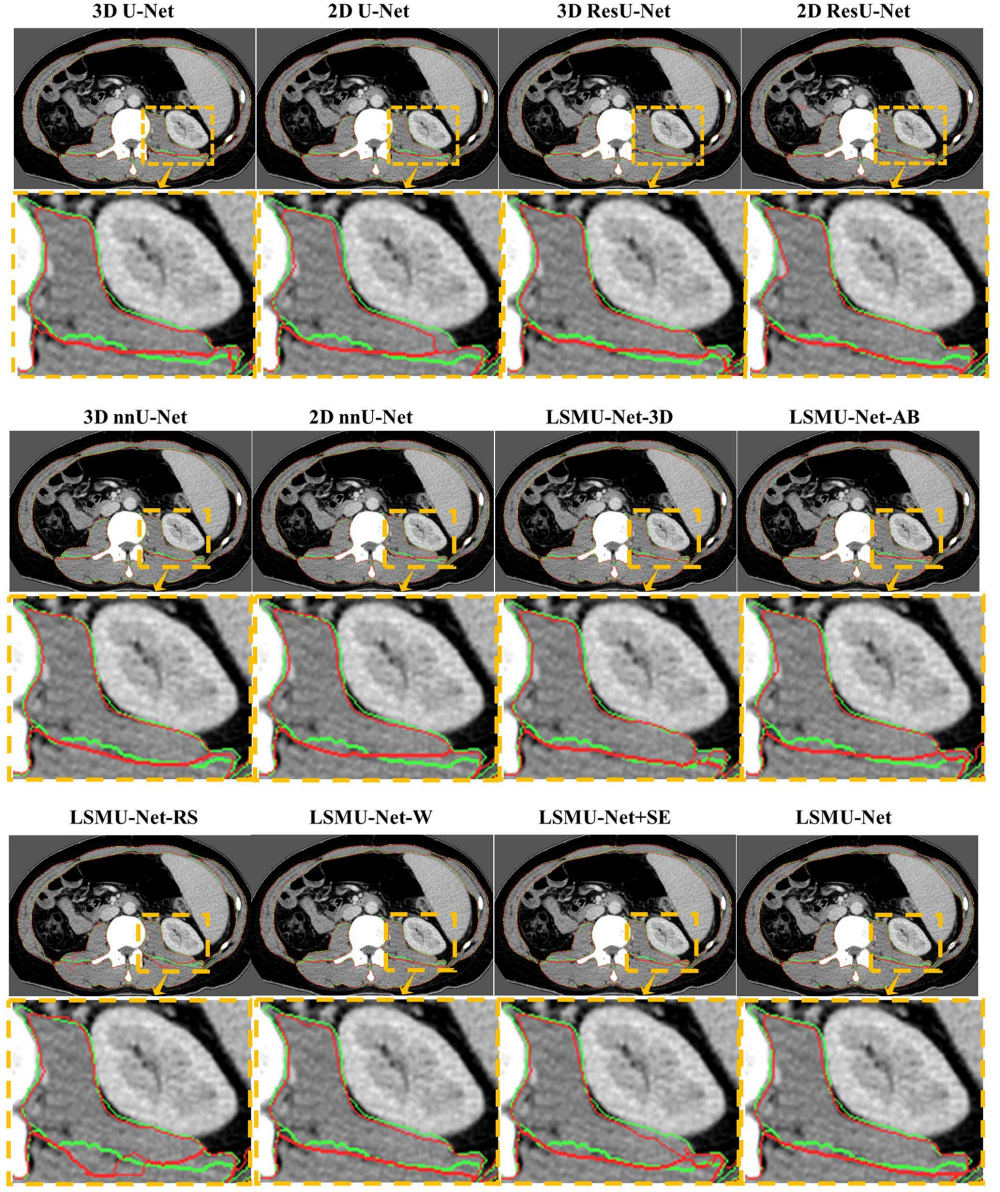
Comparison of the segmented contours and the target contours in a CT image from the generalized dataset. The green line denotes the target contour, and the red line denotes the contour of the segmentation result.

To observe whether our method achieved effective segmentation of skeletal muscle edges, Figure 4 shows the comparison of the segmented contours and the target contours in a CT image from the independent test dataset. The green line shows the contour of the target, and the red line denotes the contour of the segmentation result.

As seen in Figure 4, 2D U-Net, 3D U-Net, 3D ResU-Net, and LSMU-Net-RS have a poor effect on segmenting skeletal muscle in this data. Compared to LSMU-Net-3D, LSMU-Net-AB, and LSMU-Net-W, the LSMU-Net can reduce the wrong pixels at the edges of the segmentation results. In LSMU-Net, the green line contours overlap more with the red line contours, especially in uneven areas. While using the 2D ResU-Net, most of the muscles are well segmented, but in the magnified edge part, it is still non-fine edge segmentation compared to LSMU-Net. While compared with the network using SE attention (i.e., LSMU-Net + SE), LSMU-Net shows smoother boundary segmentation. This shows that the effect of edge refinement of the network proposed in the study is obvious. The visualization results of nnU-Net is similar to that of LSMU-Net.

**Figure 5:**
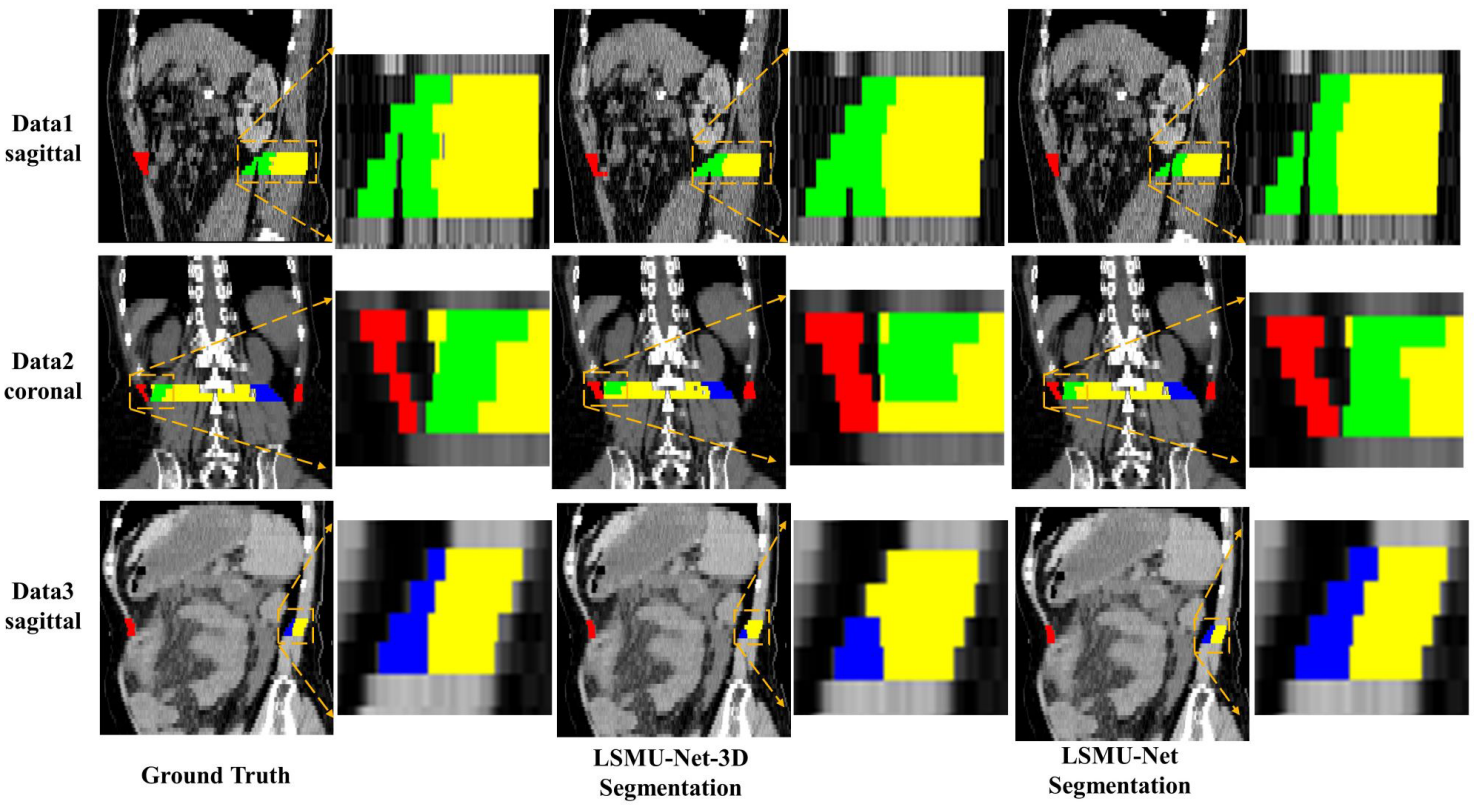
Illustration of the performance of 3D encoding branch by qualitative comparison of the segmentation results of any three CT images by LSMU-Net-3D and LSMU-Net from the sagittal or coronal views, respectively. The magnified region shows that the result output by LSMU-Net is closer to the ground truth label for the area between the right psoas (green) and the paravertebral muscles (yellow) that is difficult to distinguish.

To illustrate the performance of the 3D encoding branch, Figure 5 visualizes the results of any three CT images segmented by LSMU-Net-3D and LSMU-Net from the sagittal or coronal views, respectively. Red, green, blue, and yellow represent the segmentation of Rectus Abdominis, Right Psoas, Left Psoas, and Paravertebral, respectively. For the muscle boundary region between the Right Psoas and Paravertebral muscles that is difficult to distinguish, the segmentation result by LSMU-Net is closer to the Ground Truth label than that by LSMU-Net-3D by observing the magnified corresponding area. The reason is that the texture attention block of 3D encoding branch enhances the spatial integrity of the skeletal muscle bundle, thereby solving the challenge of identifying the boundaries of skeletal muscle bundles.

### 3.4. Auxiliary diagnostic information

As mentioned previously, the existing diagnostic index for ‘sarcopenia’ is the assessment of overall skeletal muscle (e.g., L3SMI). However, the larger the skeletal muscle volume involved in the calculation, the more reasonable the calculated value for diagnosing the presence or absence of sarcopenia. In the study, the average cross-sectional area of skeletal muscle volume corresponding to the L3 vertebra is used. Furthermore, since our LSMU-Net can segment four skeletal muscle regions in all L3-related axial slices, this makes it possible to quantitatively investigate the symptoms of cirrhotic sarcopenia in multiple muscle regions around L3. Therefore, this study will take the L3SMI, the diagnostic index of sarcopenia, as criterion to explore its relationship with the muscle indices of the four skeletal muscle regions.

The relationship was explained by the correlation analysis in the CT images of 98 patients in the cirrhosis group. Firstly, the average cross-sectional areas of the total skeletal muscle volume, as well as the four skeletal muscle regions, were calculated, respectively; secondly, referring to L3SMI’s formula, that is, the ratio of the skeletal muscle area to the square of the body height, four potential diagnostic indices were obtained, i.e., rectus abdominal index (RAI) based on Rectus Abdominis region, right psoas index (RPI) based on Right Psoas muscle region, left psoas index (LPI) based on Left Psoas muscle region and paravertebral index (PI) based on Paravertebral muscle region. Here, the total psoas index (TPI) was calculated by summing the Left Psoas and Right Psoas muscle region; finally, according to gender and whether it is sarcopenia, the correlations between the new index and L3SMI were calculated and listed in Table 4.

**Table 4:** Correlation analysis between new indices (RAI, RPI, LPI, PI, and TPI) and L3SMI in the CT images of 98 patients in the cirrhosis group according to gender and whether it is sarcopenia.

	RAI	RPI	LPI	PI	TPI
Sarcopenia	0.879	0.767	0.693	0.697	0.767
Non-sarcopenia	0.902	0.842	0.799	0.772	0.848
Female	0.935	0.712	0.692	0.862	0.766
Male	0.926	0.842	0.780	0.796	0.836
All Data	0.932	0.839	0.801	0.831	0.847

**Figure 6:**
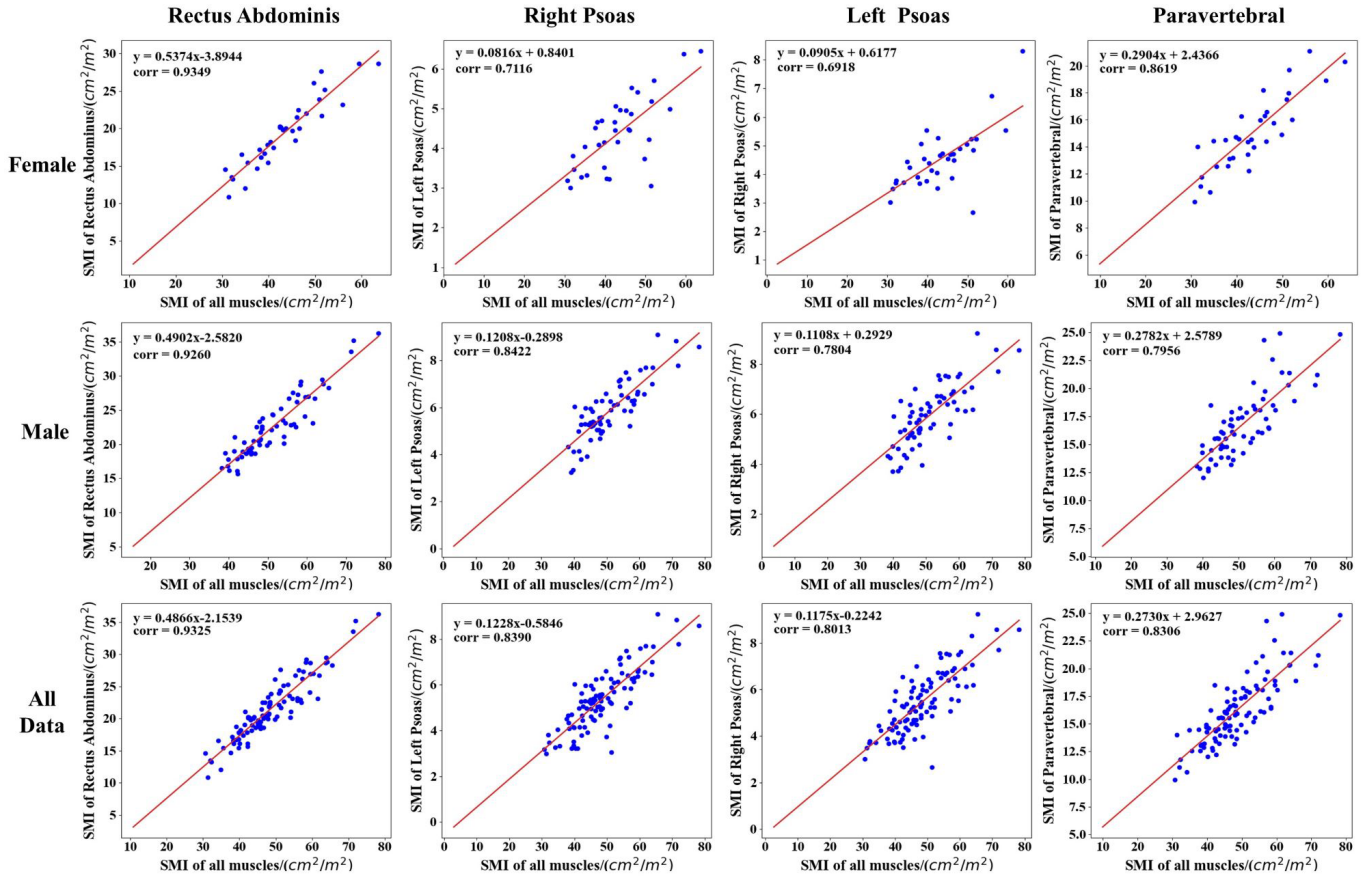
Correlation analysis between the new indicator and L3SMI according to the gender of the patients in the cirrhosis group.

Figure 6 also visualizes the correlation analysis between the new indicators and L3SMI depending on the gender of the patients in the cirrhosis group. As seen in Table 4 and Figure 6, the correlation between the five new indicators and L3SMI is higher in Non-sarcopenia patients than in Sarcopenia patients. Compared to the Male patients, the RAI and PI of the Female patients have a higher correlation with L3SMI, while their RPI, LPI, and TPI have a lower correlation with L3SMI. The correlation between RAI and L3SMI is the highest regardless of gender and whether the patient suffered from sarcopenia. From the overall data, the correlation between all indices and L3SMI is greater than 0.80.

Furthermore, according to the diagnostic cut-off value of L3SMI, Table 5 lists the cut-off value, corresponding Accuracy and AUC of the five new indicators in the diagnosis of cirrhotic sarcopenia in female and male, respectively. Due to the highest correlation between RAI and L3SMI, the diagnostic accuracy of 0.941 can be achieved by selecting the appropriate cut-off value such as 16.67 cm^2^/m^2^ in female. Therefore, the Rectus Abdominis can achieve the alternative diagnostic effect in cases where the overall skeletal muscle is not available. As seen in Table 5, the diagnostic effect of skeletal muscle region index is RAI > PI > LPI = RPI = TPI for female and RAI > LPI = TPI > RPI > PI for male.

**Table 5:** Cut-off value and corresponding accuracy of the five new indicators in the diagnosis of cirrhotic sarcopenia in female and male.

		RAI	RPI	LPI	PI	TPI
Female	Cut-off value	16.67	4.14	3.76	13.20	7.27
	Accuracy	0.941	0.794	0.794	0.882	0.794
	AUC	0.929	0.796	0.708	0.829	0.679
Male	Cut-off value	22.51	5.84	6.10	17.28	12.51
	Accuracy	0.891	0.859	0.875	0.828	0.875
	AUC	0.889	0.862	0.875	0.823	0.871

The receiver operating characteristic (ROC) curve provides a simple way to observe the diagnostic performance of a clinical indicator. The performance of the ROC curve is usually expressed by the area under curve (AUC), the value of which is the size of the area under the ROC curve. The closer the AUC is to 1.0, the higher the performance of the diagnostic index. When the AUC is equal to 0.5, the performance of the diagnostic index is the lowest. Table 5 shows the AUC of RAI, RPI, LPI, PI and TPI in females and males, respectively. It can be seen that the RAI index performed best in identifying cirrhotic sarcopenia in females and males. The diagnostic cut-off values for skeletal muscle regional indicators selected from the AUC results are ordered as RAI > PI > RPI > LPI > TPI for female and RAI > LPI > TPI > RPI > PI for male.

## 4. Conclusion

This study presented an automatic segmentation method of multi-region skeletal muscle in abdominal or abdominopelvic CT images. Our method achieved good performance by combining the appearance of skeletal muscle regions in CT images into advanced U-Net architecture. Specifically, our method includes enhancement of the existing U-Net models; texture attention enhancement block for augmenting the blurred edges of skeletal muscles; 3D encoding branch for extracting feature of muscle fiber bundles; and loss functions using the prior knowledge to reduce the class imbalance. Therefore, our method accurately segmented the multiple skeletal muscle regions from all L3-related axial slices in more than 300 abdominal or abdominopelvic CT images, and the segmentation prediction time meets the clinical real-time requirement.

Based on the segmentation results of four skeletal muscle regions, the five skeletal muscle region indices were calculated, and their correlation with L3SMI was quantitatively analyzed in the diagnosis of sarcopenia. The five skeletal muscle region indices, especially RAI, could be used to assist in the diagnosis of sarcopenia in cases where the total muscle was not available.

## 5. Discussion

Clinically, sarcopenia is usually diagnosed by L3SMI calculated on the skeletal muscle region. Existing deep learning methods have greatly improved the performance of skeletal muscle segmentation, however, for patients with cirrhosis, skeletal muscle may be squeezed and deformed by pathological changes, resulting in errors in the calculation of L3SMI. This study proposed the lumbar skeletal muscle segmentation network based on the U-Net enhanced by residual structure to segment four skeletal muscle regions in all axial CT slices related to L3 (i.e., LSMU-Net). The average cross-sectional area of four skeletal muscle regions can be used to calculate the diagnostic indexes of sarcopenia.

Comparative ablation experiments showed that the LSMU-Net method proposed in the study has good performance in terms of DSC, Sensitivity, and ASSD, which indicates the feasibility of LSMU-Net. The experimental results also showed that 2D nnU-Net and 3D nnU-Net perform well in the segmentation tasks. LSMU-Net is slightly superior to 2D nnU-Net in DSC, Sensitivity, and ASSD. LSMU-Net is slightly superior to 3D nnU-Net in ASSD and Sensitivity, while DSC is lower than the corresponding values of 3D nnU-Net. The performance of our method is achieved by combining the advanced 2D U-Net with residual structure, texture attention enhancement blocks, 3D encoding branches and the priori knowledge. Different from our LSMU-Net, nnU-Net still uses the original U-Net structure, but achieves good performance with the help of many advanced techniques, such as image preprocessing, dynamic adaptation of network topology, training strategy, inference post-processing and so on. Therefore, in addition to the improvement of network structure, the optimization and integration of data processing and training methods are also extremely important in future segmentation work.

Among the four skeletal muscle regions, Rectus Abdominis and Paravetebral muscle are larger, while Right Psoas and Left Psoas are smaller. From the perspective of segmentation evaluation index, the index of the first two regions is higher, while that of the latter two regions is slighter lower. This shows that the proposed network still has difficulties in segmenting small targets such as the Right Psoas and the Left Psoas, and the performance of the segmentation method needs to be improved.

In addition to L3SMI, sarcopenia is also diagnosed by psoas muscle index (PMI). PMI is often calculated based on the psoas major muscle, defined as the ratio of the cross-sectional area of bilateral psoas major muscles to the square of body height. The PMI’s cut-off values are 5.24 cm^2^/m^2^ in males and 3.85 cm^2^/m^2^ in females (Dolan et al., 2019). In this study, TPI was calculated based on the custom Left Psoas and Right Psoas regions, which includes the psoas major muscle and the psoas square muscle. TPI’s cut-off values are 12.51 cm^2^/m^2^ in males and 7.27 cm^2^/m^2^ in females. Obviously, TPI is defined in a larger skeletal muscle region than PMI, which may be a useful complement to PMI.

According to the results of AUC, in females, the comprehensive performance of RPI and LPI is higher than that of TPI; while in males, the diagnostic value of LPI (AUC = 0.875) is similar to that of TPI (AUC = 0.871), but different from that of RPI (AUC = 0.862). The comprehensive performance of RPI and LPI could not be compared to that of TPI in males. This issue may be related to the small number of samples of existing data sets, which need to be explored and analyzed in more cases of cirrhotic sarcopenia.

The segmented network and the five new metrics of skeletal muscle regions could better assist physicians. The results of this study may play a very important auxiliary role in the diagnosis of cirrhotic sarcopenia, especially in cases where intact skeletal muscle is not available in axial CT slices. However, this study also has some shortcomings, such as the data set is only from one institution, and the number of cases is only 317. In addition, the study only considered the effects of muscle and did not address other parameters, such as intra-abdominal fat, organ fat and subcutaneous fat. In the future, we will combine these parameters for further study to improve the automatic diagnosis of sarcopenia.

## Data availability statement

The original contributions presented in the study are included in the article/Supplementary material, further inquiries can be directed to the corresponding authors.

## Ethics statement

Written informed consent was obtained from the individual(s) for the publication of any potentially identifiable images or data included in this article.

## Author contributions

GS did the experiment and wrote the article. JZ scanned and labeled the clinical images. KW and DY reviewed the experimental process and results. SC determined the clinical patient collection plan and designed the whole plan. YS designed the whole plan, guided the experiment, and revised the article. GS and JZ are co-first authors. All authors contributed to the article and approved the submitted version.

## Conflict of interest

The authors declare that the research was conducted in the absence of any commercial or financial relationships that could be construed as a potential conflict of interest.

## Publisher’s note

All claims expressed in this article are solely those of the authors and do not necessarily represent those of their affiliated organizations, or those of the publisher, the editors and the reviewers. Any product that may be evaluated in this article, or claim that may be made by its manufacturer, is not guaranteed or endorsed by the publisher.

## Footnotes

^1^ https://github.com/MIC-DKFZ/nnUNet
