# Supplementary material for: Segmentation of multi-regional skeletal muscle in abdominal CT image for cirrhotic sarcopenia diagnosis
Source: Front Neurosci. 2023 Jun 5;17:1203823. doi: 10.3389/fnins.2023.1203823 (PMC10289291; doi:10.3389/fnins.2023.1203823)
Supplement: Supplementary file 1 [file Data_Sheet_1.docx]

Supplementary Material

Segmentation of Multi-Regional Skeletal Muscle in Abdominal CT Image for Cirrhotic Sarcopenia Diagnosis

Genshen Song*, Shiyao Chen, and Yonghong Shi

*** Correspondence:** Shiyao Chen: chen.shiyao@zs-hospital.sh.cn; Yonghong Shi: yonghong.shi@fudan.edu.cn

# Supplementary Figures and Tables

## Supplementary Figures

**Supplementary Figure 1.** Comparison of the segmented contours and the target contours in a CT image from the independent test dataset. The green line outlines the target, and the red line outlines the segmentation result.

**Supplementary Figure 2.** Comparison of the segmented contours and the target contours in a CT image from the independent test dataset. The green line outlines the target, and the red line outlines the segmentation result.

## Supplementary Tables

Supplementary Table 1. LSMU-Net ablation comparison experiment shown on Sensitivity in the independent test dataset.

|  | **#** | **3D** | **AB** | **RS** | **W** | **Rectus Abdominis** | **Right Psoas** | **Left Psoas** | **Paravertebral** |
| --- | --- | --- | --- | --- | --- | --- | --- | --- | --- |
| **3D U-Net** | 1 |  |  |  |  | 0.926±0.003 | 0.902±0.004 | 0.900±0.004 | 0.945±0.002 |
| **2D U-Net** | 2 |  |  |  |  | 0.951±0.001 | 0.930±0.001 | 0.918±0.001 | **0.964±0.001** |
| **3D ResU-Net** | 3 |  |  | ✔ |  | 0.911±0.004 | 0.914±0.004 | 0.915±0.003 | 0.952±0.001 |
| **2D ResU-Net** | 4 |  |  | ✔ |  | **0.952±0.001** | **0.952±0.001** | 0.914±0.002 | 0.949±0.001 |
| **3D nnU-Net** | 5 |  |  |  |  | 0.945±0.001 | 0.944±0.001 | 0.932±0.001 | 0.932±0.001 |
| **2D nnU-Net** | 6 |  |  |  |  | 0.942±0.001 | 0.940±0.002 | 0.929±0.002 | 0.929±0.002 |
| **LSMU-Net based** | 7 |  |  | ✔ | ✔ | 0.922±0.002 | 0.945±0.001 | 0.929±0.001 | 0.952±0.001 |
|  | 8 | ✔ |  | ✔ | ✔ | 0.943±0.001 | 0.939±0.001 | 0.926±0.001 | 0.958±0.001 |
|  | 9 | ✔ | ✔ |  | ✔ | 0.916±0.003 | 0.914±0.002 | 0.906±0.002 | 0.952±0.002 |
|  | 10 | ✔ | ✔ | ✔ |  | 0.949±0.001 | 0.942±0.001 | **0.940±0.001** | 0.954±0.001 |
| **LSMU-Net+SE** | 11 | ✔ |  | ✔ | ✔ | 0.947±0.001 | 0.950±0.001 | 0.913±0.001 | 0.946±0.002 |
| **LSMU-Net** | 12 | ✔ | ✔ | ✔ | ✔ | 0.943±0.002 | 0.941±0.001 | 0.938±0.001 | 0.953±0.001 |

**Note: #**: Method number; **3D**: 3D encoding branch; **AB**: Attention block; **RS**: Residual structure; **W**: weights

Supplementary Table 2. LSMU-Net ablation comparison experiment shown on ASSD in the independent test dataset.

|  | **#** | **3D** | **AB** | **RS** | **W** | **Rectus Abdominis** | **Right Psoas** | **Left Psoas** | **Paravertebral** |
| --- | --- | --- | --- | --- | --- | --- | --- | --- | --- |
| **3D U-Net** | 1 |  |  |  |  | 0.631±0.204 | 1.063±3.363 | 2.601±68.961 | 0.758±1.398 |
| **2D U-Net** | 2 |  |  |  |  | 0.484±0.072 | 0.884±3.055 | 0.933±5.701 | 0.480±0.031 |
| **3D ResU-Net** | 3 |  |  | ✔ |  | 0.560±0.126 | 0.974±6.547 | 0.734±1.274 | 0.497±0.094 |
| **2D ResU-Net** | 4 |  |  | ✔ |  | 0.519±0.074 | 0.744±1.081 | 0.858±2.077 | 0.444±0.037 |
| **3D nnU-Net** | 5 |  |  |  |  | **0.361±0.017** | **0.673±1.348** | **0.667±1.337** | 0.610±1.980 |
| **2D nnU-Net** | 6 |  |  |  |  | 0.370±0.016 | 1.183±12.123 | 1.260±11.275 | 0.442±0.135 |
| **LSMU-Net based** | 7 |  |  | ✔ | ✔ | 0.456±0.089 | 0.799±1.477 | 0.805±1.417 | 0.465±0.040 |
|  | 8 | ✔ |  | ✔ | ✔ | 0.439±0.053 | 1.118±12.658 | 0.733±1.506 | 0.489±0.340 |
|  | 9 | ✔ | ✔ |  | ✔ | 0.641±0.158 | 1.540±12.745 | 1.862±33.261 | 1.075±1.858 |
|  | 10 | ✔ | ✔ | ✔ |  | 0.505±0.086 | 0.943±3.585 | 0.795±1.499 | 0.464±0.194 |
| **LSMU-Net+SE** | 11 | ✔ |  | ✔ | ✔ | 0.474±0.059 | 0.771±1.526 | 0.812±1.743 | 0.436±0.082 |
| **LSMU-Net** | 12 | ✔ | ✔ | ✔ | ✔ | 0.431**±**0.045 | 0.689±1.299 | 0.701±1.409 | **0.410±0.030** |

**Note: #**: Method number; **3D**: 3D encoding branch; **AB**: Attention block; **RS**: Residual structure; **W**: weights
